# Efficient segmentation of active and inactive plaques in FLAIR-images using DeepLabV3Plus SE with efficientnetb0 backbone in multiple sclerosis

**DOI:** 10.1038/s41598-024-67130-6

**Published:** 2024-07-15

**Authors:** Mahsa Naeeni Davarani, Ali Arian Darestani, Virginia Guillen Cañas, Hossein Azimi, Sanaz Heydari Havadaragh, Hasan Hashemi, Mohammd Hossein Harirchian

**Affiliations:** 1 University of the Basque Country (UPV/EHU), Bilbao, Spain; 2 Department of Neurosciences, University of the Basque Country (UPV/EHU), Bilbao, Spain; 3 Faculty of Mathematical Sciences and Computer, Kharazmi University, Tehran, Iran; 4 Neurology Department, Imam Khomeini Hospital, Tehran University of Medical Sciences, Tehran, Iran; 5 Department of Radiology, School of Medicine, Tehran University of Medical Sciences (TUMS), Tehran, Iran; 6 Iranian Center of Neurological Research, Neuroscience Institute, Tehran University of Medical Sciences, Tehran, Iran

**Keywords:** Diseases, Medical research, Neurology, Nanoscience and technology

## Abstract

This research paper introduces an efficient approach for the segmentation of active and inactive plaques within Fluid-attenuated inversion recovery (FLAIR) images, employing a convolutional neural network (CNN) model known as DeepLabV3Plus SE with the EfficientNetB0 backbone in Multiple sclerosis (MS), and demonstrates its superior performance compared to other CNN architectures. The study encompasses various critical components, including dataset pre-processing techniques, the utilization of the Squeeze and Excitation Network (SE-Block), and the atrous spatial separable pyramid Block to enhance segmentation capabilities. Detailed descriptions of pre-processing procedures, such as removing the cranial bone segment, image resizing, and normalization, are provided. This study analyzed a cross-sectional cohort of 100 MS patients with active brain plaques, examining 5000 MRI slices. After filtering, 1500 slices were utilized for labeling and deep learning. The training process adopts the dice coefficient as the loss function and utilizes Adam optimization. The study evaluated the model's performance using multiple metrics, including intersection over union (IOU), Dice Score, Precision, Recall, and F1-Score, and offers a comparative analysis with other CNN architectures. Results demonstrate the superior segmentation ability of the proposed model, as evidenced by an IOU of 69.87, Dice Score of 76.24, Precision of 88.89, Recall of 73.52, and F1-Score of 80.47 for the DeepLabV3+SE_EfficientNetB0 model. This research contributes to the advancement of plaque segmentation in FLAIR images and offers a compelling approach with substantial potential for medical image analysis and diagnosis.

## Introduction

Various neurological disorders such as Multiple Sclerosis (MS), a complex autoimmune condition, are related to chronic inflammatory processes that result in demyelination of the nervous system^1^. For diagnostic and therapeutic management of MS, the number and volume of lesions cannot be measured using any other means^2^. Magnetic resonance imaging (MRI) is essential to characterise and quantify lesions^3^. Fluid-attenuated inversion recovery (FLAIR) and T1-weighted (T1-w) MRI techniques are currently used to diagnose MS^4^. Because MS lesions vary in location, size and shape, as well as anatomical variation between patients, MRI images of MS lesions can be extremely difficult to identify^5^. This highlights the need for more advanced diagnostic tools, such as deep learning, to accurately diagnose MS lesions.

Not every lesion seen on MRI is active^6^. Patients should be identified for treatment when enhancing lesions are considered active^7^. For this reason, Gadolinium (Gd)-based contrast agents (GBCAs) are routinely administered to MS patients during an MRI scan, as part of patient management^8^. There is a correlation between the presence of Gd-enhancing lesions and the occurrence of clinical relapses in MS^9^ which suggests that the number or volume of lesions may be an important measure of treatment efficacy^10^.

In the context of MS, MRIs have been shown to help predict future lesion activity, defined as the presence of new or enlarged T2-lesions in future images, using a Bag-of-Lesions brain representation, and to identify potential treatment responders based on carefully designed image features^11^. There has also been some success in predicting the conversion to Clinically Definite MS (CDMS) using support vector machines (SVM) on radiomic lesion features^11^ and using a CNN on lesion masks^12^. Injection of GBCAs is essential for the management of MS^8^. Repeated injections raise concerns regarding the patients' health^13^.

MS patients' Gadolinium lesions often increase, which is an indicator of disease activity^14^. The presence of contrast agent (CA) uptake indicates an early stage of inflammation and a disruption of the blood–brain barrier (BBB) in the body, allowing us to detect diseases that are in their early stages using Gd-enhanced T1w MRI^15^.

To enhance the contrast between tissues during MRI, GBCAs are used. The atomic structure of this type of substance is determined by whether it is ionic or non-ionic, or by its molecular structure. GBCAs are considered less prone to immediate hypersensitivity (IHS) reactions than iodinated contrast media (ICM)^16^.

Despite the high efficacy along with potential side effects, other alternatives for GBCAs should be considered. In the studies conducted by CA, Gadolinium was consistently shown to be retained in the bones after GBCA administration^17–20^. Gadolinium chelated in the form of gadopentetate, gadoterate, or gadodiamide has demonstrated some early evidence of bone retention in mice and rats^21^. Gadolinium is believed to retain more in bone than other tissues because it functions as a deep container or reservoir for the chemical^22^. The compound is gradually released due to osteoblast integration into the bone matrix over time^23,24^.

Histological examination reveals that rats given 80 human equivalent doses of gadodiamide developed ulcerations and fibrosis, collagen deposition, a decrease in extracellular space, dermal thickening, and an increase in cellularity^25^.

Investigating the potential of deep learning in predicting amplified lesions without injection of GBCA of MS lesions from MRI images is essential for the clinical evaluation and treatment planning of MS^26^. In this study, we do not focus on the interpretation of images, but rather on the extraction of intrinsic information from the MRI dataset. We investigate the potential of deep learning in predicting the activity of MS lesions without the use of GBCA.

Over the past decade, extensive research has been conducted in the field of cognitive rehabilitation through computer programming and specialized software tailored for individuals diagnosed with MS^27^. These studies, aimed at enhancing the quality of life for this group of individuals, have garnered significant attention^28^. The aim of this paper to propose an efficient approach for the segmentation of active and inactive plaques in FLAIR images without the need for GBCA injection, using a deep learning model based on DeepLabV3Plus SE with the EfficientNetB0 backbone. We evaluated the performance of our model on a dataset of MS patients and compared it with other state-of-the-art methods.

### Contributions

- Proposal of a novel and efficient methodology specifically designed for segmenting both active and inactive plaques in FLAIR images.
- Elimination of the need for GBCA injections, reducing patient discomfort and potential side effects associated with contrast agents.
- Utilization of a sophisticated deep learning architecture based on DeepLabV3Plus SE, augmented with the EfficientNetB0 backbone, to optimize computational efficiency and scalability while leveraging the advantages of semantic segmentation.
- Comparative analyses with several state-of-the-art segmentation methods to benchmark the performance of the proposed approach and demonstrate its superiority in terms of both accuracy and efficiency.
- Significant contribution to the field of medical image analysis by providing a robust and efficient solution for plaque segmentation in FLAIR images, facilitating more accurate diagnosis and monitoring of neurological disorders such as multiple sclerosis.

## Related work

Multiple influential research works have made substantial contributions to the field of automated lesion detection and segmentation through the application of convolutional neural networks (CNNs) and innovative architectural designs. These studies include one that explores using a CNN-based approach to detect ischemic stroke lesions in FLAIR MRI scans, enhancing accuracy by utilizing a pre-trained UNet model to pinpoint the areas affected and improve segmentation precision within axial-plane images^29,30^. Moreover, another study introduces a deep learning method for the automatic identification of ischemic stroke lesions in brain MRI FLAIR images. This employs CNN-driven segmentation and classification, providing a consistent framework for disease detection^31^. Additional research work focuses on a CNN segmentation approach aimed at extracting MS lesions from 2D brain MRI slices, significantly refining MS detection through the utilization of the Visual Geometry Group (VGG) U-Net architecture^32^.

In a related domain, a study introduces an encoder-decoder neural network used for segmenting retinal lesions in fundus images^33^. Furthermore, another research work introduces the Attention Residual U-Net model, which integrates various sophisticated features within the U-Net architecture to achieve highly accurate skin lesion segmentation^34^. Lastly, another study delves into polyp detection in colonoscopy and endoscopy images, employing a Deep Convolutional Neural Network-based (DCNN-based) methodology using the U-Net architecture to facilitate the automated detection of polyps in medical imaging^35^.

In their paper, Gamal et al.^36^ introduce a novel deep learning architecture called global attention network (GAU) U-Net specifically tailored for MS segmentation in MRI scans. The proposed GAU U-Net model draws inspiration from the widely recognized U-Net architecture, known for its effectiveness in semantic segmentation tasks, particularly in medical imaging. The GAU U-Net extends the traditional 3D U-Net by integrating a new attention mechanism inspired by the Global Attention Upsample unit. Through comprehensive evaluation, the authors demonstrate notable improvements in segmentation accuracy, as indicated by the Dice coefficient, compared to both the baseline 3D U-Net and U-Net with attention. Specifically, the Dice coefficient increases from 64 to 72% when transitioning from 3D U-Net to GAU U-Net, and from 69% to approximately 72% when comparing with U-Net with attention. Remarkably, these enhancements are achieved while reducing the number of model parameters significantly, with GAU U-Net utilizing 28 million parameters compared to 100 million parameters in U-Net with attention. This underscores the efficacy of the proposed GAU U-Net architecture for MS lesion segmentation, offering both improved performance and computational efficiency.

Rondinella et al.^37^ present a framework leveraging an augmented U-Net architecture with a convolutional long short-term memory (LSTM) layer and attention mechanism to enhance the segmentation of MS lesions in magnetic resonance images. While previous methods rely on standard architectures like U-Net, this novel approach incorporates temporal-aware features and attention mechanisms, yielding significant performance improvements. Quantitative and qualitative evaluations demonstrate superior results compared to state-of-the-art approaches, achieving an overall Dice score of 89%. Moreover, the framework exhibits robustness and generalization capabilities, validated on previously unseen samples from an ongoing dataset, underscoring its potential for automated MS lesion analysis.

Sarica et al.^38^ present a novel dense residual U-Net model enhanced with attention gate (AG), efficient channel attention (ECA), and Atrous Spatial Pyramid Pooling (ASPP) modules for precise segmentation of MS lesions from 3D MRI sequences. The proposed architecture incorporates dense connections and residual blocks within the U-Net framework, optimizing feature extraction and propagation. AGs are strategically integrated to capture salient features through skip connections, while ECA modules are employed to enhance feature representation. Additionally, ASPP is integrated to extract multi-scale contextual information crucial for accurate segmentation. Leveraging FLAIR, T1-weighted (T1-w), and T2-weighted (T2-w) MRI sequences jointly, the model achieves superior performance on the ISBI2015 and MSSEG2016 datasets, surpassing expert annotations and state-of-the-art methods. With an ISBI score of 92.75, mean Dice score of 66.88%, mean positive predictive value (PPV) of 86.50%, and mean lesion-wise true positive rate (LTPR) of 60.64% on ISBI2015, and mean Dice score of 67.27%, mean PPV of 65.19%, and mean sensitivity of 74.40% on MSSEG2016, the proposed model demonstrates remarkable efficacy in MS lesion segmentation, particularly outperforming other methods on the ISBI2015 testing set.

Automated segmentation of MS lesions from MR imaging sequences is challenging due to their diverse shapes and scattered distributions. Despite advancements in deep learning, existing methods struggle with capturing scattered lesions and delineating global contours accurately. To address these issues, Chen et al.^39^ proposed DAG-Net, a deep attention and graphical neural network that integrates spatial correlations and global context. Their approach utilizes a novel local attention coherence mechanism for dynamic graph construction and a spatial-channel attention module for enhancing feature representation. Experimental results on benchmark datasets demonstrate DAG-Net's effectiveness in segmenting variant and scattered MS lesions, highlighting its potential for improving automated MS lesion segmentation.

Joshi and Sharma^40^ present a novel hybrid network that combines a convolutional neural network (CNN) autoencoder with graph convolution networks (GCN) for MS lesion segmentation. This approach leverages 3D medical resonance image (MRI) voxels as nodes in a graph dataset, with the CNN autoencoder extracting imaging grid information and GCN learning features in the graph connectivity space. Trained and validated on datasets from MS patients, the hybrid network demonstrates improved segmentation performance, achieving a dice similarity coefficient score of 85.5% for three neighbors in the graph data. This research showcases the potential of integrating CNN and GCN for advancing automated MS lesion segmentation techniques.

Bouzidi et al.^41^ present a novel approach for MS lesion detection using magnetic resonance imaging (MRI). They treat tumor segmentation as a classification problem and employ the Ant Colony Optimization algorithm (ACO) combined with BrainSeg3D tools. Their study evaluates this approach on a longitudinal database of 20 MS patients, comparing results with ground truth annotations and other methods like Dissimilarity Map (DM) creation. This research contributes to improving MS lesion detection methods, crucial for effective disease management.

Together, these studies collectively underscore the advancement and practical application of sophisticated neural network architectures in automated lesion detection and segmentation, significantly propelling progress in medical imaging and disease diagnostics.

The diagnostic process in the aforementioned researches is notably sensitive and challenging due to the large size and heightened vulnerability of lesions in the nervous system in the studied diseases, along with the relatively limited dispersion of these lesions within the brain. In fact, in patients with MS, lesions appear extremely small and scattered in MRI images, significantly complicating their identification^42^. Nonetheless, our research, employing advanced methodologies, has managed to identify lesions in MS patients with the highest precision and efficiency even under such challenging circumstances. This achievement underscores the enhanced potency and impact of our utilized methodology in intricate and demanding conditions like those presented in cases of MS.

## Materials and methods

The main goal of this study was to use a CNN model for segmenting active and inactive plaques in FLAIR images. In the following, we explain the different pre-processing methods used to improve the dataset before the main processing, and then the best model for this problem is introduced. Finally, the model EfficienNetB0, which is used as the backbone of this study, will be introduced.

Furthermore, meticulous and accurate labeling was a paramount focus in this study, ensuring that no alterations or changes were introduced to the images during the labeling process. This deliberate approach was adopted to mitigate the risk of labeling errors and enhance the precision of the results.

The utilization of five Nvidia 3090 graphics cards proved to be of great significance in this investigation. These graphics cards, renowned for their high processing power, provided the computational capability required for complex and extensive data analysis. This computational prowess greatly contributed to the research's efficiency and overall success.

Additionally, the emphasis on precise and unaltered labeling within this research holds substantial importance. In image processing and deep learning studies, labeling accuracy is a critical aspect. The decision to refrain from making any modifications to the images during the labeling process was made to minimize the potential impact of errors in labeling, ultimately leading to more accurate research outcomes.

### Dataset

This research focused on analyzing a cross-sectional cohort of 100 patients diagnosed with MS who exhibited active plaques in their brains. In this study, a total of 5000 MRI slices were investigated. This comprised approximately 25 FLAIR slices and 25 T1 slices with gadolinium injection, totaling 50 slices (including T2 FLAIR and T1 with gadolinium) for each patient. After filtering the images based on the presence of active or inactive plaques, 1500 slices were used in the labeling and deep learning process.

The proposed model was exclusively fed with flair images of patients, where their corresponding labels depicted a black background. Active and inactive plaques were distinguished with white (pixel value 255) and gray (pixel value 127) colors, respectively.As a result, T1 images were omitted from the network's input. However, their labels were still employed in training the model for active plaque detection, aligning with the model's specifications and needs.

The labeling process in this article was performed using the Pixlr Suite program, Pixlr was founded in 2008 and built on Macromedia Flash. Prior to the labeling process, lesions in all three axial, sagittal, and coronal dimensions were confirmed by a radiologist. The labels were applied to the lesions in a manner that enables their selection and enhancement using the advanced tools of this software. It is worth mentioning that a pen was not used in the labeling process to minimize errors. Active plaques were compared with reference images, while inactive plaques were also assessed for comparison.

### Pre-processing

To improve the data, dataset pre-processing was done. It was employed to improve the data's compatibility with subsequent steps. Removal of the cranial bone segment of the skull, resizing, and normalization were the pre-processing steps carried out in this study. In the following sections, a thorough explanation of various pre-processing steps is provided.

#### Removal of the cranial bone segment

In the preprocessing stage, to eliminate the surrounding cranial bone segment of the skull from our images, could after applying the 3 × 3 median filter, use a combination of the Gabor filter and morphological operation.

The Gabor filter used in this section is obtained from the following formula^43^:

1 $$g\left(x,y;\lambda ,\theta ,\Psi ,\sigma ,\gamma \right)=\text{exp}\left(-\frac{{x}^{{^{\prime}}^{2}}+{\gamma }^{2}{y}^{{^{\prime}}^{2}}}{2{\sigma }^{2}}\right)\text{cos}\left(2\pi \frac{{x}{\prime}}{\lambda }+\Psi \right),$$

where

$${x}{\prime}=x\text{cos}\theta +y\text{sin}\theta , {y}{\prime}= -x\text{sin}\theta +y\text{cos}\theta ,$$

and λ is the wavelength of the sinusoidal component whose value is equal to $5\frac{\pi }{4}$, also θ represents the orientation of the normal to the parallel stripes of the Gabor function and is considered to be equal to $1\frac{\pi }{4}$, Ψ represents the phase offset of the sinusoidal function and is set to be -0.5. σ is the standard deviation of the Gaussian envelope whose value is 1, and finally γ is the spatial aspect ratio and specifies the elasticity support of the Gabor function, which is equal to 1.

**Figure 1:**
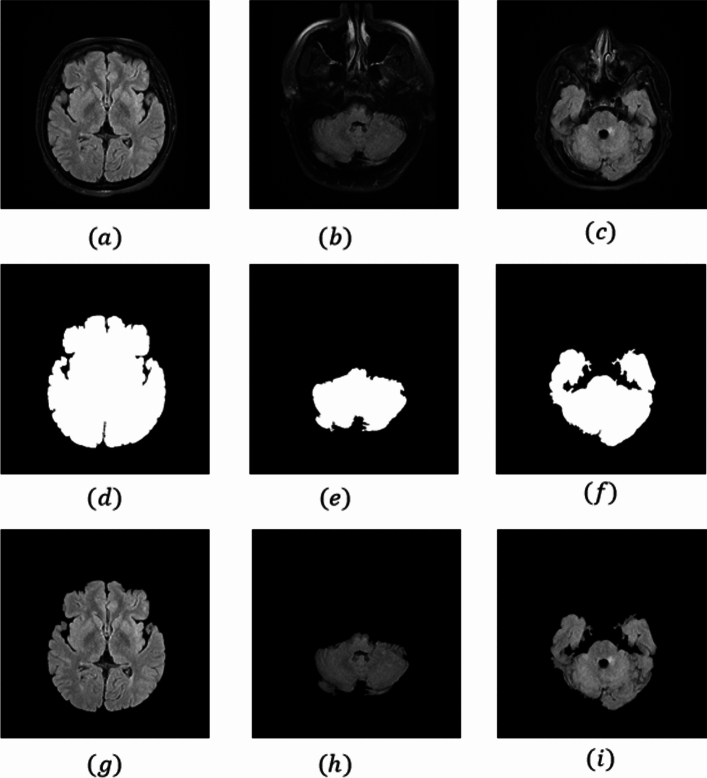
Steps to prepare images for model training. (**a**–**c**) the original flair image of the brain, (**d**–**f**) Creating a mask related to brain parenchyma tissue, and (**g**–**i**) the removal of the cranial bone segment of the skull from flair images.

After applying the Gabor filter by these parameters and morphological operators to the image, thus separating the brain parenchyma tissue from the rest of the image, and our images were ready for training the target network. Figure 1 shows all these steps for three images in general.

#### Resizing

Changing all of the images in the dataset to a specific size is referred to as resizing. We needed to resize the dataset because the neural network only receives images that are the same size. The image is 256 * 256 * 1 in size after the resizing step.

#### Normalization

To convert all of the image's pixel values between 0 and 1, a process known as normalization was used. Each pixel value is divided by 255 to accomplish this.

### Proposed method

#### Squeeze and excitation network (SE-Block)

**Figure 2:**
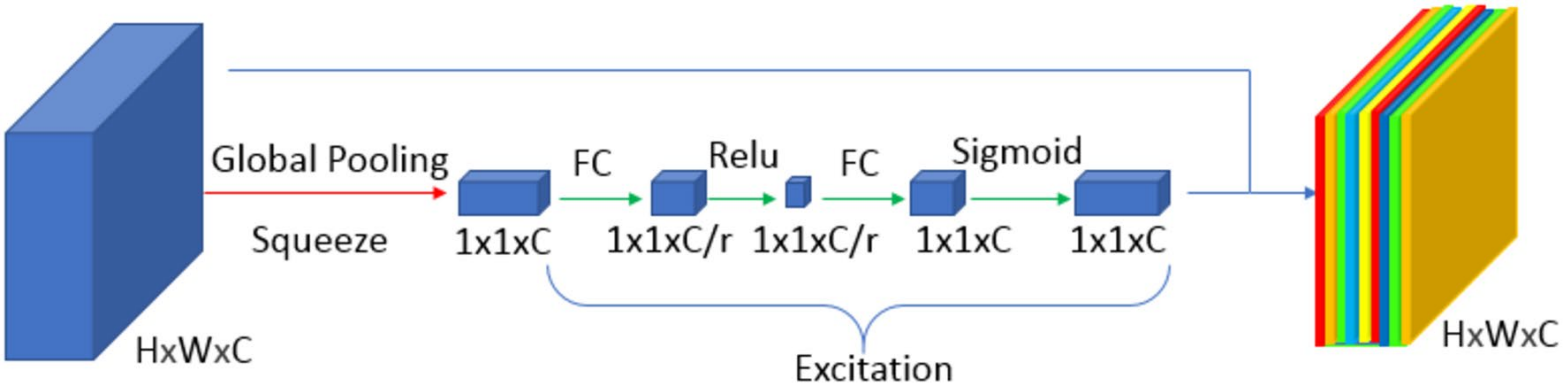
Structure of Squeeze-and-Excitation Net (SENet). The red arrow represents the squeeze stage, the blue arrow represents the scale stage, and the green arrow represents the excitation stage.

The SE block, shown in Fig. 2, focuses on important feature maps and suppresses less important feature maps^44^. This improves the model's ability to identify the target object in the input image, potentially resulting in better segmentation results. The SE block is added after using three convolutional excitations with two activations, Relu, and a sigmoid activation function.

#### EfficientNetB0

**Figure 3:**
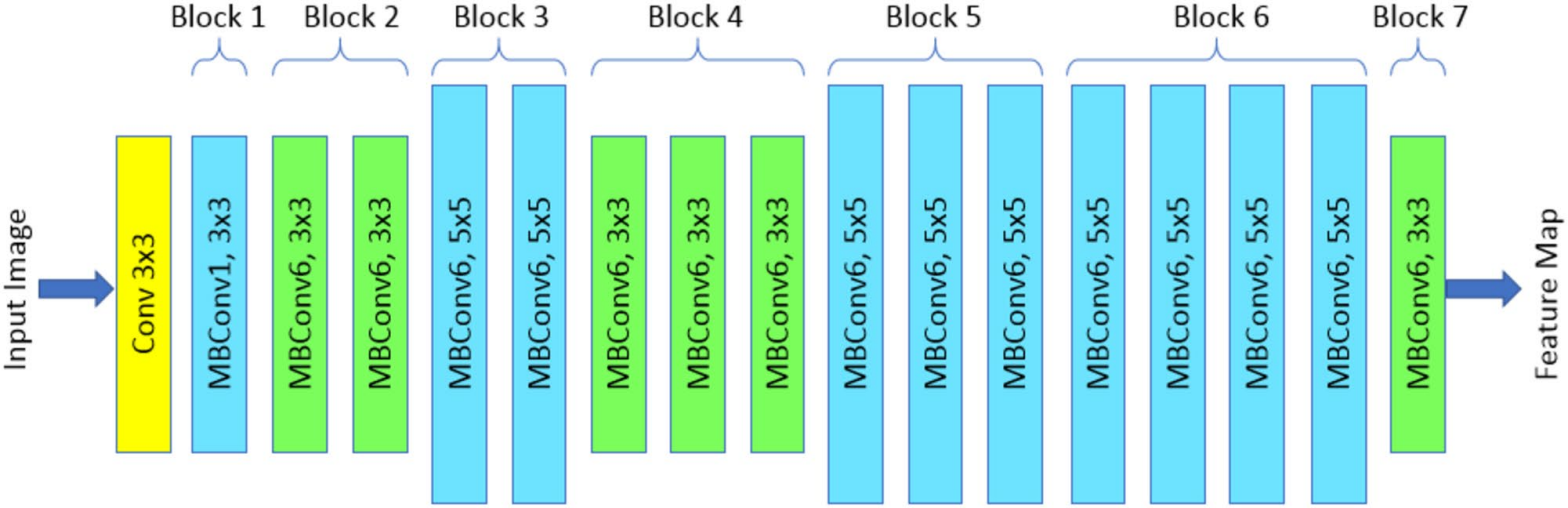
Architecture of EfficientNet-B0.

The convolutional neural network design and scaling method known as EfficientNetB0 uniformly scales all depth, width, and resolution measurements using a composite factor^45^. The EfficientNet scaling method uses a current set of scaling constants to continuously expand the width, depth, and resolution of the network, unlike traditional scaling methods that scale these components arbitrarily. The basic EfficientNetB0 system is based on MobileNetV2's inverse bottleneck residual block in a collection of squeeze blocks and excitation blocks^46^. The EfficientNetB0 Architecture, shown in Fig. 3.

#### Atrous spatial pyramid pooling (ASPP) block

The ASPP method is designed to capture multi-scale context information between encoder and decoder and is especially used for semantic segmentation^47,48^.

However, instead of using basic convolution (typically rate = 1) and max pooling or average pooling in blocking of Deeplabv3plusSE, we used ASPP modules with different rates^1,6,12,18^ depth and point convolution (basic convolution) instead, reducing computational complexity. The formula for the ASPP module is:

2 $$\left[i\right]= \sum_{k}x\left[i+r , k\right]w[k],$$

**Figure 4:**
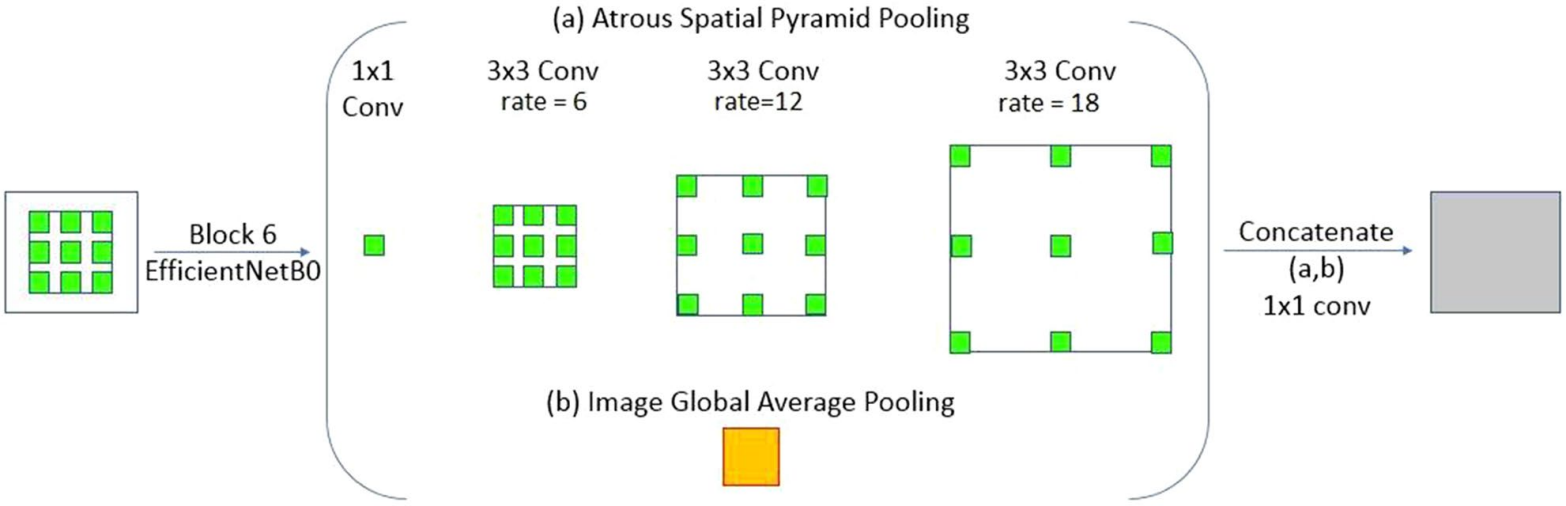
Structure of the ASPP module used in DeeplabV3+SE. This module consists of two stages, including (**a**) Atrous convolution and (**b**) Image Pooling, and produces the final output using a convolution layer after concatenating the feature maps.

Here, the steps required to sample the input feature map are denoted by the associated rate, r. x and w are the input signal and filter, respectively. When r = 1, the basic standard convolution becomes a special instance of the separable Atrous convolution. It is clear that Atrous convolution allows the kernel of deep CNN to have a wider field of view. The ASPP block is shown in Fig. 4. Supports small fields of view for accurate Region of Interest (ROI) localization and context absorption without increasing parameters or calculations due to the introduction of zeros between filter values.

Figure 4 shows that ASPP blocks with sets containing dilation and convolution kernel sizes are used at the entrance of the network. ASPP was implemented on the input of the network on the high-level image features of the sixth layer of EfficientNetB0 (our selected pre-trained model for this study) to capture the multi-scale characteristics of different plaque sizes. Details of the ASPP blocks used include four expansion factors^1,6,12,18^ with kernel sizes^1,3,3,3^, and Concatenation included (1 × 1 normal convolution).

#### Deeplabv3Plus SE network structure

**Figure 5:**
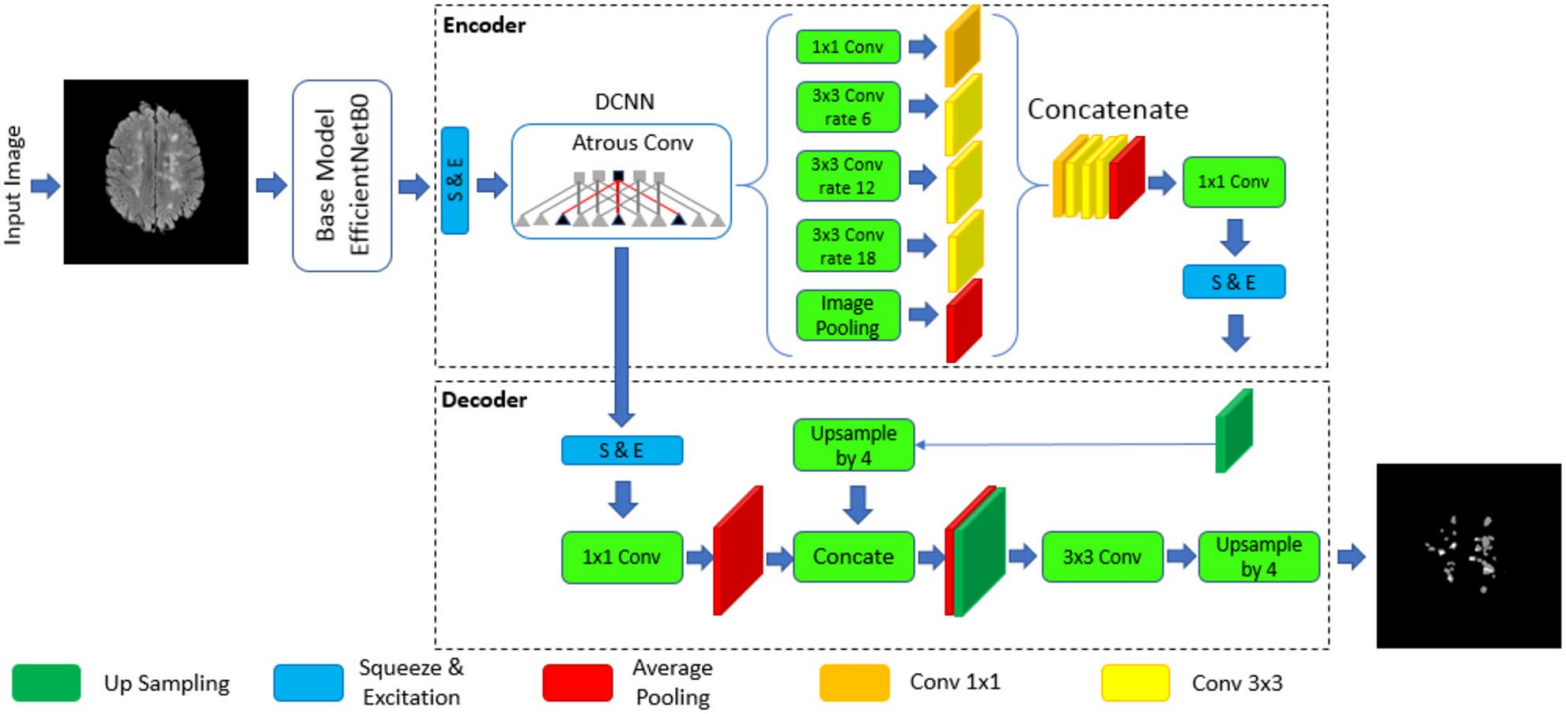
The image illustrates DeepLabv3's components: the encoder, a pre-trained convolutional neural network like EfficientNetBO, extracts features from input images, while the decoder refines these features to generate a segmentation map showing the likelihood of each pixel belonging to specific classes. In the context of fingerprint recognition, these classes might represent features like ridges and valleys. The encoder uses atrous convolution to capture multi-scale information while preserving resolution, while the decoder uses upsampling, concatenation and convolution to refine and generate the segmentation map. The structure of the DeeplabV3+SE (proposed method), DCNN stands for deep convolutional neural network and Atrous Conv stands for atrous convolution.

The overall structure of the Deeplabv3Plus SE network is shown in Fig. 5. Deeplabv3Plus SE extends Deeplabv3 with a simple but effective decoder module to refine segmentation results, especially along object boundaries^49^. It consists of two parts: an encoder and a decoder. Encoders are mainly used to extract features and reduce the dimensionality of feature maps. The decoder is mainly used to recover edge information and feature map resolution to obtain the final semantic segmentation result^47^. To increase the receptive field and maintain the resolution of the feature map, the convolution operations in the last few convolutional layers of the encoder are replaced with Hall convolutions. The Atrous Spatial Pyramid Pooling (ASPP) module, introduced in DeeplabV3+SE, uses dilated convolutions at different rates to obtain multiscale semantic context information. By using these new structures, DeeplabV3+SE provides accurate semantic segmentation results across different data sets^48^.

The code use in this research is accessible via the following link: https://github.com/hosseinazimi0213120/DeepLab-V3Plus-SE-EfficientNetB0.

### Train model

After separating the parenchyma tissue of the brain from images, we utilize the DeeplabV3+SE architecture, which combines its convolution layers with the squeeze and excitation network in this research. Since the performance of a segmentation model is directly related to the selected loss function, we used the dice coefficient as the loss function. Adam's optimization was also utilized as a model optimizer. Each convolutional layer has 128 filters. Finally, the activation function was considered as a Relu activation function. In the input of the model, we used the 6th layer of EfficientNetB0 to select the high-level features of the image, and the third layer of EfficientNetB0 to select the low-level features of the image. It is worth mentioning that in transfer learning methods, models previously trained on other datasets are used to extract features. In these models, they feed the raw data to one of the pre-trained models and take the output of the convolution layers as the extracted features.

In this research, we used the pre-trained EfficientNetB0 model and used the output of the sixth convolution layer of this architecture to extract high-level features of raw images and the output of the third convolution layer to extract low-level features. Finally, we entered the image containing the extracted features as the input of the Deeplabv3Plus SE network into the model.

### Ethical approval

This research adheres to all ethical considerations relevant to the content of this article. The study was conducted in compliance with established ethical standards, and all necessary approvals and permissions were obtained from the appropriate ethical review boards. In accordance with ethical guidelines, informed consent was obtained from all participants involved in the study. The confidentiality and anonymity of participants have been rigorously protected throughout the research process.

Furthermore, the research design and procedures were ethically reviewed to ensure that they met the highest standards of integrity and transparency. Any potential conflicts of interest were appropriately disclosed, and steps were taken to mitigate and manage them.

This article discusses the ethical implications of the research, addressing key considerations such as the treatment of human or animal subjects, data confidentiality, and potential biases. The methodology employed in this study aligns with ethical principles, and any deviations from standard ethical practices are explicitly justified.

In summary, this research article upholds a commitment to ethical conduct in all aspects of the study, and the following sections elaborate on specific ethical considerations within the context of the presented research.

Code of ethics obtained from **Tehran University of Medical Sciences**:

**IR.TUMS.NI.REC.1401.038**

•We have sought approval for the study from the ethics committee of Tehran University of Medical Sciences (Imam Khomeini Hospital) in Tehran.

## Results

The results of the study prove highly beneficial for MS patients, marking a substantial advancement in medical imaging methodologies. The dataset utilized in this study comprises a considerable number of active and inactive plaques in each image slice. This contrasts with some other datasets used in different studies, which lack such diversity, resulting in a scarcity of analyzable samples for better model training. Furthermore, the plaques within these images are of notably small volume and area, sometimes encompassing less than ten pixels, leading to more errors in our model's identification of these smaller plaques due to the high precision required in the pre-processing and labeling stages.

Moreover, the discussion thoroughly evaluates the outcomes obtained from various neural network architectures, including DeepLabV3+_resnet50, DeepLabV3+_resnet101, DeepLabV3+_DenseNet121, DeepLabV3+_VGG16, Deeplabv3+_PSPNetSE_SegModel, and our proposed method, DeepLabV3+SE_EfficientNetB0. The comparison based on the Dice Score exhibited the superior performance of DeepLabV3+SE_EfficientNetB0, scoring the highest among the evaluated architectures with a Dice Score of 76.24.

To evaluate the proposed model, we first consider 20% of images as validation data, then we use six different criteria to demonstrate the performance of finding the best architecture for our study, and finally show the performance of selected backbones for our network. Calculating the IOU index: The Intersection over the union index is the overlap area between the predicted segmentation and the ground truth divided by the joint area between the predicted segmentation and the ground truth. The formula for calculating the Jaccard index is:

$$ {\text{Inter}}\sec {\text{tion}}\;{\text{Over}}\;{\text{Union}}\;{\text{index}} = \left( {{\text{overlapping}}\;{\text{region}}} \right)/\left( {{\text{joining}}\;{\text{region}}} \right). $$

Calculating DSC (Dice Score): Dice Score provides a measure of similarity between the predicted and actual segmentation masks. Its value varies between 0 and 1. 0 means no overlap, and 1 means complete overlap. The Dice Score is calculated using the following formula:

$$ {\text{Dice}}\;{\text{Score}} = \left( {2*{\text{overlap}}\;{\text{area}}} \right)/{\text{total}}\;{\text{area}}. $$

Calculating precision and recall and F1 score: Precision and recall measurements are used together to indicate the quality of the segmentation. If the precision and recall values of one segmentation result are both higher than the second, this indicates a higher quality segmentation. The F1 score is the harmonic mean between the precision and recall scores. To calculate these three criteria, proceed as follows.

$$ \Pr {\text{ecision}} = \left( {{\text{TP}}} \right)/\left( {{\text{TP}} + {\text{FP}}} \right) $$

$$ {\text{Recall}} = \left( {{\text{TP}}} \right)/\left( {{\text{TP}} + {\text{FN}}} \right) $$

$$ {\text{F}}1\;{\text{score}} = 2*\left( {{\text{precision}}*{\text{recall}}} \right)/\left( {{\text{precision}} + {\text{recall}}} \right) $$

Calculation of the Matthews Correlation Coefficient (MCC): MCC is a more reliable statistical relationship that scores highly only when the prediction performed well in all four categories of the confusion matrix (true positives, false negatives, true negatives, and false positives) , compared to the size of positive and negative elements in the dataset. The MCC is calculated using the following formula^50^:

$$MCC= \frac{TP\times TN-FP\times FN}{\sqrt{(TP+FP)(TP+FN)(TN+FP)(TN+FN)}}$$

In this study, in general, 25 neural networks from different articles were studied and implemented on this dataset, and the output of all networks can be found in the Supplementary file number 1 (sorted by dice score). Table 1 shows only the results of the top 6 models. In Table 1, we compared the performance of six architectures DeepLabV3+_resnet50, DeepLabV3+_resnet101, DeepLabV3+_DenseNet121, DeepLabV3+_VGG16, DeeplabV3+_PSPNetSE_SegModel and DeepLabV3+SE_EfficientNetB0 (our proposed method). Table 2 provides insights into the performance of all models examined in this study.

**Table 1:** CNN Architecture and Metrics in this study.

Data	CNN architecture	Metrics					
		IOU	Dice Score	Precision	Recall	F1-Score	MCC
validation	DeepLabV3+_resnet50	0.6739	0.7233	0.8564	0.6879	0.7629	0.6438
	DeepLabV3+_resnet101	0.6745	0.7291	0.8519	0.6912	0.7631	0.6681
	DeepLabV3+_DenseNet121	0.6629	0.7172	0.8409	0.68	0.7519	0.6824
	DeepLabV3+_VGG16	0.6840	0.7383	0.8710	0.7161	0.7859	0.6905
	DeeplabV3+_PSPNetSE_SegModel	0.6935	0.7595	0.8812	0.7238	0.7947	0.7144
	DeepLabV3+SE_EfficientNetB0	0.6987	0.7624	0.8889	0.7352	0.8047	0.717

**Table 2:** Provides a comparison between our study and previous research efforts. This allows us to evaluate the performance of our model in relation to existing methods.

	References	Purpose	Datasets	Methods	Limitations	Results
1	Ava Assadi Abolvardi et al.^53^ (Australia)	Registration Based Data Augmentation for Multiple Sclerosis Lesion Segmentation	Longitudinal MS lesion dataset	3D-UNET/V-NET	Not reported	Dice score = 0.61.4
2	MOSTAFA SALEM et al.^54^ (Spain)	Multiple Sclerosis Lesion Synthesis in MRI Using an Encoder-Decoder U-NET	Dataset1: The ISBI2015 dataset includes 181 (axial and sagittal) and 217 (coronal) slices. Dataset2: (15 healthy and 65 patients MS (Vall d'Hebron hospital, Barcelona	A CNN Model	Ability to control the intensity and the texture inside the lesions and the requirement of ground-truth masks for obtaining the lesion model	Dataset1: Dice score = 0.64, Sensitivity = 0.57, precision = 0.79. Dataset2: Dice score = 0.70, Sensitivity = 0.69, precision = 0.73
3	Shahab Aslani et al.^55^ (Italy)	Multi-branch convolutional neural network for multiple sclerosis lesion segmentation	Dataset1: The ISBI2015 dataset includes 181 (axial and sagittal) and 217 (coronal) slices. Dataset2: NRU dataset	2D end-to-end convolutional network based on the residual network (ResNet)	1.Observed that the proposed pipeline is slightly slow in segmenting a 3D image since segmenting whole-brain slices takes a longer time compared to other CNNbased approaches. 2. Computational complexity	Dataset1: Dice score = 0.61, PPV = 0.89, VD = 0.45 and Dataset2: Dice score = 0.66, PPV = 0.80, VD = 0.33
4	Yushan Feng et al.^56^ (USA)	A SELF-ADAPTIVE NETWORK FOR MULTIPLE SCLEROSIS LESION SEGMENTATION FROM MULTI-CONTRAST MRI WITH VARIOUS IMAGING SEQUENCES	The ISBI2015 dataset includes 181 (axial and sagittal) and 217 (coronal) slices	Optimized 3D U-Net with Non-uniform Patches	Not reported	Dice score = 0.68,Jaccard = 0.53, PPV = 0.78
5	Rehan Afzal et al.^57^ (Australia)	Automatic and Robust Segmentation of Multiple Sclerosis Lesions with Convolutional Neural Networks	Two datasets ISBI and MICCAI	A CNN Model	If two lesions are very close or overlapping, sometimes the proposed algorithm is unable to segment precisely. Also, when lesions are near the cortex of the brain it was difficult to segment them	ISBI Dataset: Dice score = 0.67, Precision = 0.90 and MICCAI Dataset: Dice score = 0.72
6	Florian Raab et al.^58^ (Germany)	A multimodal 2D Convolutional Neural Network for Multiple Sclerosis Lesion Detection	The MICCAI 2016 MSSEG Lesion Segmentation Challenge dataset and the ISBI 2015 Longitudinal MS Lesion Segmentation Challenge dataset	A 2D convolutional neural network, based on the U-Net	Not reported	ISBI Dataset: Dice score = 0.77 and MICCAI Dataset: Dice score = 0.69
7	Abhilasha Joshi and Sharma^40^ (India)	Hybrid Topology of Graph Convolution and Autoencoder Deep Network For Multiple Sclerosis Lesion Segmentation	Ljubljana White Matter MS Dataset(LWMMS): 3D MRI of 30 MS patients and Ljubljana longitudinal MS Dataset(LLMS): Another arrangement of MRI data of 20 MS patients	Graph convolutional network (GCN)	Limitations: 1. when neighbor increases memory for graph data to process in learning also increases which took more compilation time and space. 2. increasing the number of neighbor corporate overwrite information for graph learning	Dice score = 0.76, Precision = 0.87, Loss = 0.23
8	Dalenda Bouzidi et al.^41^ (Tunisia)	Ant Colony Optimization with BrainSeg3D Protocol for Multiple Sclerosis Lesion Detection	The images were acquired on a 1.5 T PhilipsMRI machine at the University Medical Centre Ljubljana (UMCL)	Semi-automated outliers segmentation tools of BrainSeg3D software	It is necessary to test this interface on larger databases to clear the strengths and limitations	Dice score = 0.76
9	Reda Abdellah Kamraoui et al.^59^ (France)	DeepLesionBrain: Towards a broader deep-learning generalization for multiple sclerosis lesion segmentation	ISBI dataset and MICCAI2016 MS Challenge Dataset	DeepLesionBrain	Not reported	Dice score = 0.64, PPV = 0.88, Hybrid score = 0.74
10	Zhanlan Chen et al.^39^ (Australia)	Deep Attention and Graphical Neural Network for Multiple Sclerosis Lesion Segmentation From MR Imaging Sequences	The ISBI2015 dataset includes 181 (axial and sagittal) and 217 (coronal) slices	Proposed DAG-Net is built in an encoder-decoder architecture, which is mainly comprised of the LAC mechanism and SCGA module	Not reported	Dice score = 0.65, PPV = 0.88, VD = 0.39
11	Gamal et al.^36^ (Egypt)	Multiple sclerosis segmentation	Open-MS dataset contains; 30 patients (23 males and 7 females)	Combination of Global Attention Up sample (GAU) and Unet	Not reported	Dice Coefficient = 0.72
12	Rondinella et al.^37^ (Italy)	Boosting multiple sclerosis lesion segmentation through attention mechanism	The ISBI2015 dataset includes 181 (axial and sagittal) and 217 (coronal) slices	Combining Unet, Squeeze-and-attention and LSTM	Limited number of samples available	Dice score = 0.84, IOU = 0.74, Sensitivity = 0.84 and Specificity = 0.99
13	Sarica et al.^38^ (Turkey)	A dense residual U-net for multiple sclerosis lesions segmentation from multi-sequence 3D MR images	The ISBI2015 dataset includes 181 (axial and sagittal) and 217 (coronal) slices and The MSSEG2016 dataset is composed of 3D MR images of 53 MS patients	Unet modification (combination of Dense Residual U-Net with AG, ECA, and ASPP)	Not reported	Dice score = 0.66, ISBI score = 0.92, PPV = 0.86 and VD = 0.38

**Figure 6:**
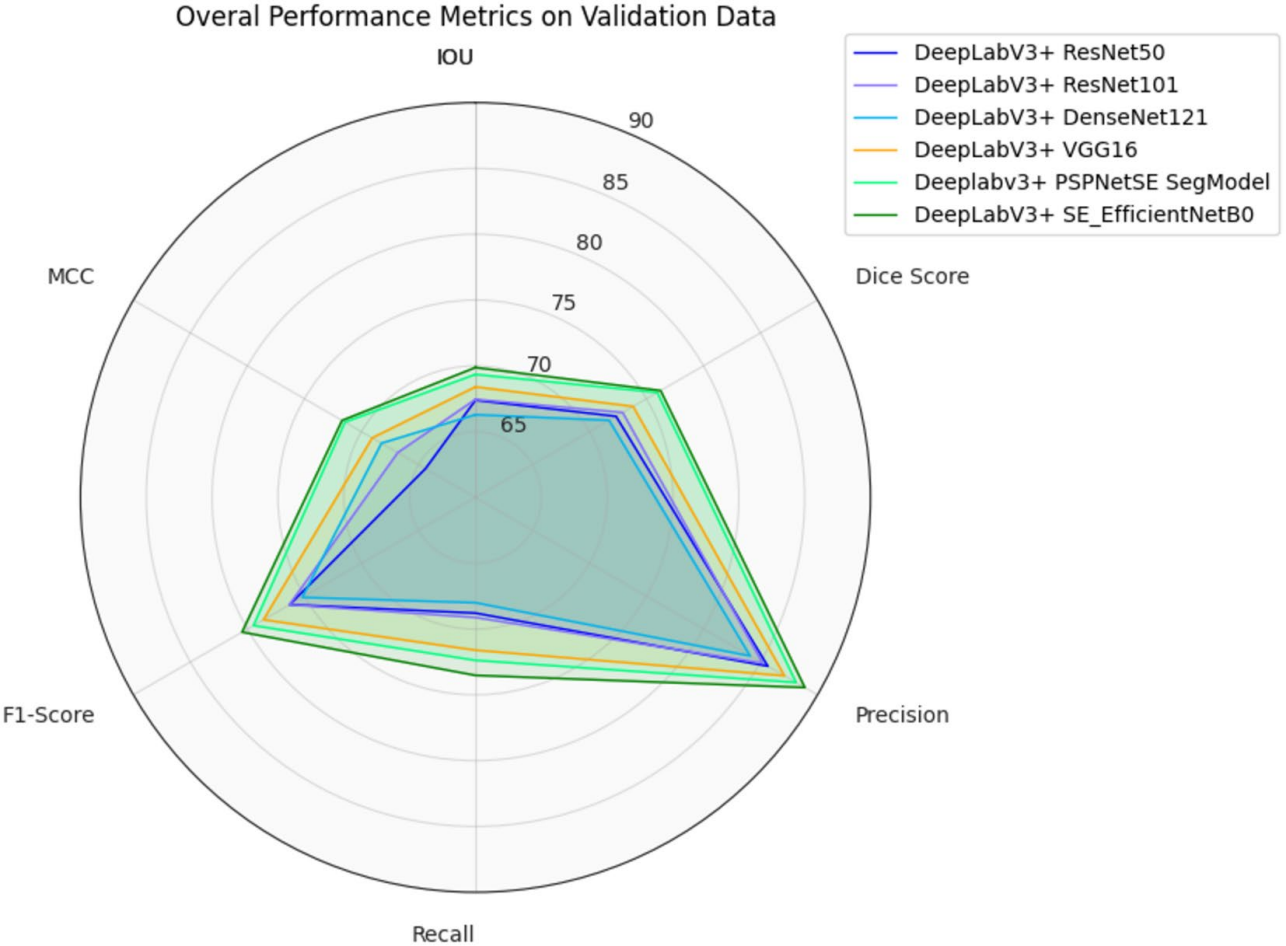
This radar plot shows the performance of six of the best models used in this study for our metrics.

According to Table 1, we used the network introduced in this study for our work, because it shows the good performance evaluation criteria of that network for the segmentation of active and inactive plaques. The results of the networks used are shown in Fig. 6.

**Figure 7:**
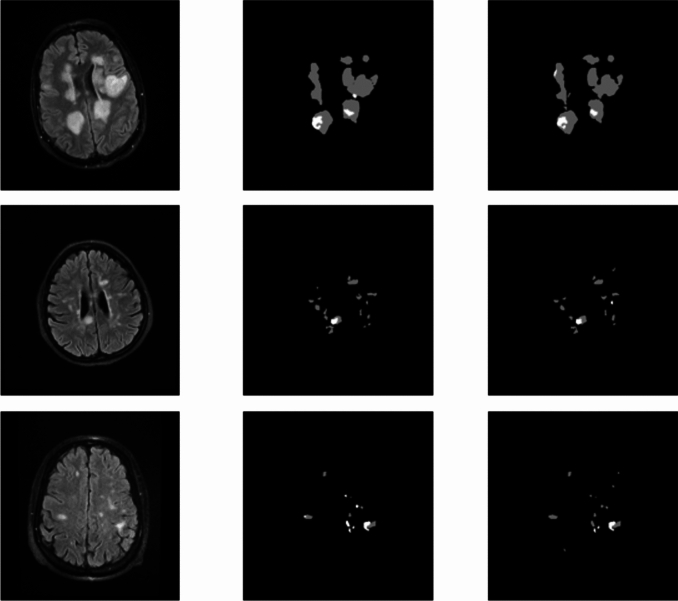
Original images (left column), ground truth associated with test samples (middle column), and images predicted by the proposed model (right column).

Furthermore, the comprehensive assessment of the DeepLabV3+SE_EfficientNetB0 model's outcomes in segmenting active and inactive plaques within FLAIR images revealed its exceptional proficiency. The metrics IOU, Dice Score, precision, recall, F1-Score, and MCC highlighted the model's exceptional ability in identifying and categorizing these plaques, outperforming other neural network architectures. In Fig. 7, we can see the performance of the proposed method for some test samples.

The current article has excelled in pioneering a novel methodology in the domain of MS patients. It has exhibited the most promising predictions and outcomes. The DeepLabV3+model, extensively utilized in various other studies and research articles, has found applications in diverse areas, ranging from automatic brain tumor segmentation^51^ to Random Region Matting for High-Resolution PolSAR Image Semantic Segmentation^52^. Nonetheless, its implementation in this particular study targeting MS patients has shown unparalleled performance and the most favorable predictions, signifying a groundbreaking advancement in the field.

## Discussion

The novel approach aimed to eliminate Gadolinium injection by introducing advanced analytical tools in artificial intelligence and innovative algorithms, addressing the limitations of traditional diagnostic techniques associated with side effects. This innovative approach not only enhances diagnostic accuracy but also potentially mitigates the health risks linked to conventional contrast agents.

## Conclusion

In conclusion, the integration of this model in future research within medical imaging is highly recommended. The potential applications of this technology in various medical and biomedical studies offer promising avenues for the development of improved disease diagnostic solutions. These solutions could play a pivotal role in the diagnosis and treatment of diseases. This innovative approach not only enhances diagnostic accuracy but also potentially reduces the health risks associated with traditional contrast agents, signifying a significant advancement in the field of medical imaging.

It is recommended for future research to examine potential correlations within their datasets, which may include sagittal and coronal cuts, and consider incorporating additional cuts for plaque segmentation. The constraints encountered in this research are outlined as follows: The network performance deteriorates significantly when dealing with images containing very small plaques, rendering it practically incapable of detection. In images where plaque structures resemble doughnut shapes (i.e., with empty centers), our model exhibits low accuracy and tends to identify the inner portion as part of the plaque, leading to errors. (However, this occurrence is quite rare.) Detection becomes somewhat challenging for our model in images where plaques extend to the outer boundaries of brain tissue or adhere closely to the contours of adjacent butterfly-shaped structures. Nevertheless, it should be noted that errors are not pervasive in such cases.

### Supplementary Information

Supplementary Information.

## Data Availability

The datasets generated and/or analyzed during the current study are not publicly available due to patient privacy concerns but are available from the corresponding author upon reasonable request.
