# Supplementary material for: Efficient segmentation of active and inactive plaques in FLAIR-images using DeepLabV3Plus SE with efficientnetb0 backbone in multiple sclerosis
Source: Sci Rep. 2024 Jul 15;14:16304. doi: 10.1038/s41598-024-67130-6 (PMC11251059; doi:10.1038/s41598-024-67130-6)
Supplement: Supplementary file 1 — Supplementary Information. [file 41598_2024_67130_MOESM1_ESM.docx]

**Twenty-five different networks were implemented, including the mentioned methods as well as several others, on our proprietary dataset. Only the top 5 in terms of accuracy were included in our report.**

| Dice Score | Model Name | NO |
| --- | --- | --- |
| 0.5811 | **PSPNet ResNet50** | **1** |
| 0.5841 | **Unet** | **2** |
| 0.5950 | **DC Unet** | **3** |
| 0.6029 | **Unet+** | **4** |
| 0.6186 | **VGG19 Unet** | **5** |
| 0.6260 | **GCN EfficientNetB0** | **6** |
| 0.6274 | **VGG16 Unet** | **7** |
| 0.6409 | **MultiRes Unet** | **8** |
| 0.6509 | **GCN ResNet50 v2** | **9** |
| 0.6653 | **MobileNetv2 Unet** | **10** |
| 0.6755 | **DeepLabV3plus SqueezeNet9** | **11** |
| 0.6794 | **InceptionResNetV2** | **12** |
| 0.6829 | **EfficientNetB0 Unet** | **13** |
| 0.6865 | **MobileNet Unet** | **14** |
| 0.6918 | **DeepLabV3plus DenseNet201** | **15** |
| 0.6933 | **DeepLabV3plus DenseNet169** | **16** |
| 0.7002 | **Xception Unet** | **17** |
| 0.7047 | **DeepLabV3Plus Resnet152** | **18** |
| 0.7125 | **DeepLabV3plus VGG19** | **19** |
| 0.7172 | **DeepLabV3plus DenseNet121** | **20** |
| 0.7233 | **DeepLabV3Plus Resnet50** | **21** |
| 0.7291 | **DeepLabV3Plus Resnet101** | **22** |
| 0.7383 | **DeepLabV3plus VGG16** | **23** |
| 0.7595 | **Deeplabv3plus PSPNet SqEx SegModel** | **24** |
| 0.7624 | **DeepLabV3plusSE EfficientNetB0** | **25** |
